# The Supply Chain’s Role in Improving Animal Welfare

**DOI:** 10.3390/ani3030767

**Published:** 2013-08-14

**Authors:** David Harvey, Carmen Hubbard

**Affiliations:** School of Agriculture, Food and Rural Development, and Centre for Rural Economy, Newcastle University, Newcastle upon Tyne, NE1 7RU, UK; E-Mail: carmen.hubbard@ncl.ac.uk

**Keywords:** farm animal welfare, supply chains, Bayesian Belief Networks, consumer-citizen gap, animal welfare standards

## Abstract

**Simple Summary:**

The ability of supply chains to deliver high(er) levels and standards of animal welfare is subject to two critical conditions: (a) the innovative and adaptive capacity of actors in the chain to respond to society’s demands; (b) consumers actually buying animal-friendly products. Unless citizens are willing to support suppliers who comply with high(er) standards, their votes for better animal welfare risk exporting poor animal welfare to other countries with less rigorous standards. The logic of market failure in the case of animal welfare points to the superiority of consumer subsidies over producer subsidies to deliver improved animal welfare.

**Abstract:**

Supply chains are already incorporating citizen/consumer demands for improved animal welfare, especially through product differentiation and the associated segmentation of markets. Nonetheless, the ability of the chain to deliver high(er) levels and standards of animal welfare is subject to two critical conditions: (a) the innovative and adaptive capacity of the chain to respond to society’s demands; (b) the extent to which consumers actually purchase animal-friendly products. Despite a substantial literature reporting estimates of willingness to pay (WTP) for animal welfare, there is a belief that in practice people vote for substantially more and better animal welfare as citizens than they are willing to pay for as consumers. This citizen-consumer gap has significant consequences on the supply chain, although there is limited literature on the capacity and willingness of supply chains to deliver what the consumer wants and is willing to pay for. This paper outlines an economic analysis of supply chain delivery of improved standards for farm animal welfare in the EU and illustrates the possible consequences of improving animal welfare standards for the supply chain using a prototype belief network analysis.

## 1. Introduction

The animal product supply or marketing chains are critical in determining and delivering animal welfare. No matter what citizens may say or governments require, if consumers do not support and reward improved standards of animal welfare, then the supply and marketing chains cannot be expected to deliver them [1]. Napolatino *et al.* [2] define and document a set of processes through which food supply chains can be described as having moved from what they portray as a vicious circle towards a more virtuous circle. The vicious circle identifies a cycle of squeezed farm margins and increased competition leading to lower animal welfare. The virtuous circle is seen as being driven by more informed consumers and by mobilizing their *willingness to pay* (WTP) for improved animal welfare so as to reward the supply chains for their efforts to ensure such improvements. 

**Figure 1:**
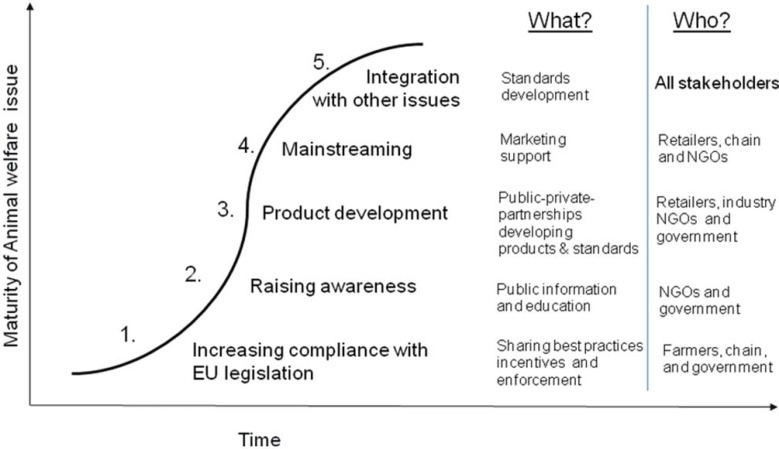
Animal welfare development road [3].

An original aim of the EU EconWelfare project, as is implicit in the title, was to estimate the economic impact (costs and benefits) of upgrading animal welfare standards on the EU supply chain and its competitiveness. However, as the project progressed, it rapidly became apparent that this aim cannot be met other than through very specific and highly conditional illustrative case studies. The bulk of the project output consisted of a comprehensive documentation of existing initiatives and supply chain developments towards higher standards and an intensive stakeholder engagement programme to identify the most promising policy instruments to assist further development without compromising the competitiveness of EU supply chains. Overall, the project identified a possible ‘road’ towards improved animal welfare within the EU, based on extensive stakeholder consultation and review of existing initiatives, as shown in Figure 1, which exhibits the classic adoption S-curve. This outline of the potential and actual progression towards improved animal welfare identifies the key elements of informing and mobilizing consumer preferences from a baseline of compliance with basic legislation (1), through raised awareness (2), to new product development (3), which generates products associated with improved welfare, which might then become the mainstream product lines (4), subsequently (or perhaps simultaneously) to be integrated with other issues such as sustainability, environmentally friendly and local production (5). 

This paper highlights the major implications of this general progression towards improved animal welfare for the supply (or marketing) chain of animal products, based on the workshops and stakeholder engagement activities pursued under the EconWelfare project. More specifically, it concentrates on the economics of the supply or marketing chain: the connections between those who deliver animal welfare (the supply side) and those who demand or require particular standards of animal welfare (the demand side). To do so, we employ a Bayesian Belief Network, under the assumption that both consumers and citizens (the demand side) as well as producers and processors (the supply side) can be sufficiently well informed about real animal welfare conditions to take the appropriate action to improve animal welfare. Whether or not either consumers or producers choose to care enough to be well informed, and whether or not they alter their behaviours as a consequence are the economic issues arising in the supply or marketing chain.

The paper is organised as follows. Section 2 gives a short overview of the typical economic framework for considering the potential effects of improving animal welfare, while Section 3 highlights the critical conditions on which further development of higher standards in the supply chains depend. Although these sections draw heavily on [1], their outline repetition here is necessary to understand the background for the empirical work outlined in Section 4. This section documents the development of a prototype belief network which can, in principle, identify the broad consequences of improved animal welfare standards in the EU. A pre-condition for the relevance of a belief network approach is that participants broadly accept a common conceptual framework which identifies the major causal relationships determining animal welfare and competitiveness. Some concluding remarks and policy recommendations are presented in Section 5. 

## 2. The Basic Economic Framework for Animal Welfare

**Figure 2:**
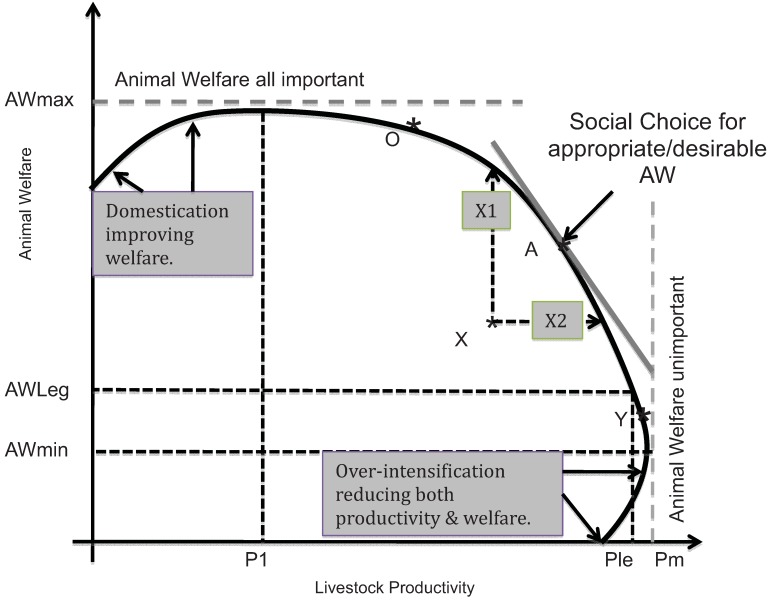
Possibilities for improving animal welfare (following [4]).

Competitive marketing or supply chains can be expected to deliver the levels and standards of animal welfare which society demands, subject to important conditions discussed below. As businesses in the supply chain continually seek to improve their commercial performance in competition with their peers and rivals, the supply chain effectively defines a *production possibility frontier (PPF)—*or industry best practice*—*which traces out the trade-offs between animal welfare and livestock productivity (production and marketing costs) as shown in Figure 2. 

Majewski *et al*. [5] document in detail the cost consequences at the farm level of a range of stylized standards, specifying higher AW standards as collections of specific norms relating to animal conditions and management practices. For all the standards examined, with the exception of dairy cows, the higher standards generated increased net costs (net of the benefits achieved by adopting the norms). In the dairy sector, at least in some countries, the research conducted by the EconWelfare team suggested that the typical dairy farm is actually currently X-inefficient in terms of Figure 2, and able to improve animal welfare and reduce production costs (improve productivity) simultaneously. Given the recent competitive pressure on dairy farms in the EU, it is to be expected that such improvements will happen as the sector adjusts towards the more efficient operations.

The implication is that competitive supply chains generally find that improvements in animal welfare are associated with reduced animal productivity (and associated increased production costs). Of course, not all firms in the supply chain are performing up to industry best practice, so some—perhaps many—will not be operating on this frontier of best practice at any given time, but will lie somewhere inside it (at a point like X). These less efficient and effective firms could improve both animal productivity and animal welfare at the same time (moving to the segment A, between X1 and X2 on the production possibility frontier). In principle, the continual processes of supply chain competition can be expected to encourage these currently inefficient firms to move closer to the PPF for animal welfare. Without this continual competition for market share and margins over costs, the pursuit of improved animal welfare is likely to be slow. In addition to competition within the supply chain, the more research and development (R&D) and effective information dissemination and extension services are available, the more rapidly such best practices can be expected to percolate through the industry, and the more rapidly can the best practice frontier be expanded (shifted upwards and to the right in Figure 2).

However, the more consumers and their representatives (the food retailers) are willing to pay for improved animal welfare, the more care the chain actors can be expected to take to deliver at least consumer/retail perceptions of improved welfare. Alternatively, the greater the restrictions imposed on the domestic marketing chains through regulation and legislation, in the interests of animal welfare, the more attention the marketing chain actors are obliged to take of animal welfare—focusing on compliance with the minimum required welfare (AWLeg) at the expense of maximum possible animal productivity (Pm). 

## 3. Critical Supply/Marketing Chain Conditions

The critical conditions determining the ability of the supply chain to deliver animal welfare can be divided into two conceptually distinct components: (a) the innovative and adaptive *capacity* of the chain to respond to society’s demands for improved animal welfare, which determines the position of the PPF, and the extent to which all firms are at or close to the industry best practice (the PPF); (b) the extent to which consumers are indeed *willing to pay* for improved animal welfare, which determines the position on the PPF towards which the supply chains will tend to adjust.

### 3.1. Capacity

There is a very limited literature on the capacity and willingness of supply chains to deliver what the consumer wants and is willing to pay for. Conventional economic analysis typically assumes that competitive pressures and the continual struggle of firms and businesses to survive and prosper will be sufficient to ensure that ‘gaps in the market’ will be found by enterprising individuals and businesses. It also supposes that any gaps which are not filled result from the practical problem that the costs of meeting and filling the gap are not sufficiently recompensed by the sales to make the enterprise worthwhile—there is no market in the gap.

Franz *et al.* [6] report on an action‐based analytical approach which seeks to identify different barriers within the supply chain that prevent the establishment of a market segment for animal welfare products, using the German market as a case study. These authors appear to be motivated by the observation that “*the market offer of animal welfare products is clearly lower than consumer demand*” ([6], p. 319). However, it should be noted that the documentation for this assertion includes many references which relate to attitudes and hypothetical WTP studies, which are subject to the problems and issues discussed below, and which cannot be taken as *prima facie* evidence of a genuine gap in the market. In addition, these researchers note that the problems and issues they address are essentially short-term or transition/adjustment issues, rather than fundamental long-term or permanent barriers, consistent with the PPF picture above.

Marketing premium, animal friendly products requires that the supply chain is appropriately segmented, differentiated and traceable, which is typically more costly than marketing chains which ignore origins, processes, and provenance of products. While these tracing and differentiation costs are typically prohibitive for chains that are not already differentiated, recent development of food markets has led many chains to become substantially differentiated for reasons other than simply animal welfare. Notwithstanding the substantial concentration in the slaughtering, processing and retailing sectors, there is already a large number of differentiated and separately labeled animal product lines, at least in the richer western European countries, which can be further elaborated with improved animal welfare provisions as and when demand warrants it. This progression is illustrated in Figure 1 above, as the evolution of animal product markets towards point 5, as societies become both richer and better informed. Possibly the greatest barrier to further differentiation, or enhancement of existing labels with additional animal welfare provisions, is *consumer information overload* ([6], p. 319).

Major retailers (especially) are continuing to differentiate their own label product lines, emphasizing quality, origins and production systems. As already with organic labels, these premium and quality products are likely to become more animal welfare friendly as retailers recognize the threats to their brands and corporate images should media stories emerge about insensitive treatment of animals in their supply chains. Franz *et al.* [6] appear convinced by their supply chain participants (in Germany) that: “*Most products are marketed using price arguments instead of emphasizing specific qualities. Especially in the meat industry the perception is widely spread that there are only small niches for brands and specialty products. Therefore, price competition, i.e., the cost leadership strategy clearly dominates the meat industry* (p. 323). However, the proliferation of premium and differentiated brands over the past two decades suggests that this characterization is both too simple and also now rather out-of-date (see [7] for details and extent of differentiation).

Since there is a clear and well-founded resistance to developing brands and labels which solely relate to animal welfare, the evidence and logic points to an approach which integrates animal welfare with other dimensions of quality, and packages animal welfare with these other dimensions [8]. These authors analyse the well-established proposition that the introduction and imposition of industry-wide ‘minimum quality standards’ (MQS) in an already differentiated and segmented market does not necessarily improve average animal welfare or social welfare. In particular, successively higher imposed MQS can ‘crowd out’ private (voluntary) premium initiatives, not only directly but also indirectly as firms adapt their competitive strategies. As they point out ([8], p. 274/5), since the mid 1990s European retailers have introduced a number of initiatives aimed at differentiating their higher welfare standards and product quality from their competitors. These private agreements on sets of process standards are generally more demanding than the public MQS, and are subject to regular monitoring by third-party certifiers. An important part of the marketing strategy is making it clear and (reasonably) transparent to consumers that the products are of higher quality than required by the public standard. These developments, and the reputations of suppliers and retailers associated with the delivery of reliable quality products, effectively negate the theoretical arguments about ‘markets for lemons’ [9]. This theory depends on asymmetric information, where suppliers are supposed to know far more about the provenance of their products than consumers, and hence have an apparent incentive to pass off low quality (poor animal welfare products) as if they were high quality. The recent horsemeat scandal illustrates that this theoretical possibility is strongly subject to market responses as and when the ‘lemons’ are exposed.

Codron *et al*. [8] also neatly summarise the conflicting incentives facing retailers for the appropriate level of the public MQS. On the one hand, retailers would like the standard to be set as high as possible, to avoid their own costs associated with differentiating their own products and segmenting their own markets—in effect, passing responsibility for the quality and safety of their products to the public sector (and the suppliers). On the other hand, the confidence, trust and loyalty of consumers is of vital importance, and they cannot afford to either betray this confidence to a public sector which may not be up to the job or relinquish market share to competitors who may well be able to obtain lower quality products with less provenance from other (overseas) sources. *In particular, they show that overall social welfare (the sum of the interests of producers, consumers and taxpayers) is higher under a combination of mandatory public MQS at a relatively basic level coupled with differentiation and segmentation of private labels and standards.* Social welfare is reduced if the public MQS is set too high, particularly since the incentives to cheat and avoid the standards (and consequent diminishing trust by consumers in the imposition and meaning of the standard) are greater the higher the level of the MQS. Furthermore, and not discussed by Codron *et al.*, setting MQS at a high level will tend to reduce the range of product choice available to consumers and limit the potential for continued adoption of more welfare friendly products in the future. 

While supply chain barriers are frequently asserted to mitigate the development of socially responsible supply chains, perhaps especially for improved animal welfare, two major factors should be noted. First, supply chains are continually evolving and adapting to changing conditions, especially the willingness of consumers to recognise and pay for quality and differentiation of food products, including improved animal welfare. The progress made in the past is testament to this continual change—relatively few people appear to believe that animal welfare conditions are deteriorating, while the majority thinks that conditions have improved [10]. Market trends confirm that more people are now more willing to pay the necessary premia for improved (or at least differentiated) quality than in the past. Notwithstanding the present economic downturn, there is no substantial reason to suppose that these trends will not continue. Second, even the most sophisticated and mature food supply chains remain capable of further improvement, and can be expected to strive to further improve their operations and co-ordination as competition for resources, inputs and market shares continue to tighten. Researchers (e.g., [11]) continue to develop approaches and frameworks through which supply chain relations, interactions and transactions can be improved to better reflect consumer and citizen demands and requirements to primary producers.

The EU EconWelfare project has identified and examined a wide variety and large number of initiatives throughout the EU. These demonstrate both the capacity of at least parts of the supply chain to deliver improved animal welfare and also its ability and willingness to participate in public-private partnerships and collaborations to further improve animal welfare conditions throughout the chain. A major outstanding difficulty relates to the apparent gap between citizens’ aspirations and ambitions for improved animal welfare and their willingness to pay for such improvements. 

### 3.2. The Citizen/Consumer Gap

There has been both a growth in more animal welfare friendly product lines and chains as societies grow richer and better informed (Figure 2), and an increasing number of scientific surveys of consumers’ willingness to pay (WTP). These appear to demonstrate significant to substantial premia available for improved welfare products [1]. Nevertheless, there is a continued belief that people vote for substantially more and better animal welfare as citizens than they are willing to pay for as consumers. Improved animal welfare products continue to be seen as niche markets, while animal welfare is typically regarded as a public good—once ensured for one citizen or consumer it is ensured for all (technically, non-rival in consumption). As a result, it is frequently argued that governments should take responsibility, since citizens’ votes (on moral grounds) should surely take precedence over consumers’ WTP (based on self interest). 

However, citizens do not abdicate responsibility for their moral judgments (votes) by delegating responsibility to governments. Unless citizens are willing to support their local suppliers in compliance with higher standards, their votes for better animal welfare may amount to nothing more than cheap talk. Even vegetarians and vegans exert market influence: by purchasing nothing to do with animals, they remove their dependence on farm animals and reduce the number needed (at whatever level of welfare) to satisfy the demand. The overall level of animal welfare in any society is, in principle, some sum of each animal’s level of welfare over the whole population of animals. The overall level of animal welfare, therefore, depends on the consumer demands for the final products, otherwise the animals would not be kept, at whatever level of welfare. Animal products, and hence their associated levels of welfare, are not public goods, they are rival in consumption. Notwithstanding the ultimate moral or philosophical foundations for our individual and collective concerns with animal welfare (which are clearly both complex and contested), as and when we individually and collectively decide to act on these beliefs, and determine that our animals are better treated, then we need to be prepared to bear the associated costs of doing so. Whatever the moral or philosophical bases of our beliefs and animal welfare preferences, we have to acknowledge and accept responsibility for the economic costs of exercising these preferences. Neither markets nor governments can be expected to meet the aspirations of citizens if these citizens themselves are not willing to provide the necessary supportive actions to achieve these aspirations. 

As Harvey and Hubbard [1] explore and explain in more detail, there are six good reasons to expect citizens’ expressions (votes) in favour of improved animal welfare to appear to be substantially stronger than their apparent WTP for more products of higher animal welfare standards. Only one of these reasons is ‘cheap talk’—people not being willing to put their money where their mouths are, for whatever underlying and no doubt complex reasons. Another potentially important reason is the ‘free-rider’ problem associated with the psychological consumption externality involved in peoples’ judgments about animal welfare. The externality arises as an effect on my wellbeing which is generated by your consumption (and hence by the associated production), rather than an actual *public good* [12]. Nevertheless, my consumption of products from poor animal welfare production systems helps to perpetuate these poor systems and adversely affects others who value animal welfare more highly than I do. If I can be persuaded not to consume these products, these other people will feel better off. I, too, might be more prepared to switch my consumption towards more animal welfare friendly products (even if at higher cost) if I could be sure that other people would also do so. But if not, there is a strong temptation for me to consider that my own efforts in favour of better animal welfare are too small to make any substantial difference and hence not worth my effort. If everyone else also succumbs to this selfish temptation, then society as a whole will end up with less animal welfare than they really want (and are willing, at least in principle, to pay for). 

These consequences are termed the “*free-rider*” problem, which is the principal reason for government intervention. As Harvey and Hubbard [1] demonstrate, markets fail if the free-rider deficit is greater than the market deficit—where the free-rider deficit is defined as the shortfall of expressed WTP from the total WTP presuming that there is no free-rider problem, while the market deficit is the additional costs of ensuring the higher welfare standard through the supply chains. If the free-rider deficit is not sufficient to offset the market deficit, then it is not socially optimal to require or encourage the delivery of the higher standard, and there is no market failure [13]. The problem-consistent solution to the free-rider problem is to subsidise the consumption of animal welfare friendly products—adding an *ex-gratia* top-up to each Euro spent on approved higher standard animal welfare products—rather than to subsidise production, which is the typical intervention. As Harvey and Hubbard [1] argue at greater length, consumption subsidies are not only self-limiting, since they will only be paid if consumers actually purchase higher animal welfare products, but also specifically encourage the development of higher welfare standard supply chains and are also much more consistent with international trading obligations and agreements than a producer or production subsidy (*c.f.* [16]). 

In practice, however, there are four other major reasons why consumers might not be willing to pay as much for improved animal welfare as their responses as citizens appear to indicate. Two of these are unreliable labels and inadequate information. Both can and should be countered through supply chain actions, providing that the additional returns warrant the costs. However, the provision of reliable information itself is a public good—once supplied for one, it is effectively supplied for everyone, and my use of a given bit (or byte) of information does not deny you the use of the same bit. There is a strong case for government (public) provision and support of such reliable information (including the third party verification of labels and marketing claims, and of R&D and extension of best practices).

Nevertheless, two final reasons for the gap between citizen votes and consumer spending in favour of higher animal welfare strongly suggest that there will always be such a gap, and that it may well be quite wide. These two reasons are: (a) that it takes too much effort to check the information available and read the labels; (b) that there is too much else to worry about and put effort into, and these things are considered by many consumers to be more important or more urgent than animal welfare. These two reasons are at the root of most of the so-called ‘hypothetical bias’, which plagues attempts to measure WTP. The focus of specific opinion polls (or sophisticated choice experiments to measure WTP) on the issue of animal welfare in isolation from the rest of increasingly complex lives and livelihoods is quite likely to result in answers which differ substantially from real world practice and behaviour. While general education might be expected to improve the salience of animal welfare to people, and hence increase their likelihoods of caring more and taking more care about animal welfare, it is to be expected that there always will be some gap between citizens’ votes and consumers’ WTP which is inevitable and inherent in the human condition, and not necessarily an indication of either market or regulation failure.

## 4. A Bayesian Belief Network: Chain Consequences of Improved Standards for Animal Welfare

As noted in the introduction, it is impossible to identify a simple and meaningful calculation or illustration of the impact of upgraded standards of animal welfare on the EU supply chain and its competitiveness. This impossibility is a reflection of the complexity of interrelationships between the characteristics and efficacy of specific on-farm and off-farm welfare standards, costs and benefits, farm structures, characteristics and efficiency of the chain, and welfare outcomes. As a proxy, we develop an outline representation of the consequences of imposing upgraded standards throughout the distribution chain using a causal Belief Network (BN) of the potential consequences of upgrading standards [17,18]. Belief networks provide a natural and efficient method for graphically representing probabilistic dependencies and causal relationships among a set of variables. In essence, they represent causal linkages in a system as a ‘directed graph’, which shows how particular variables (e.g., market demand) influence others (such as animal welfare) in a causal manner. Belief networks also provide consistent semantics for representing uncertainty and an intuitive graphical representation of the interactions between various causes and effects. As a consequence, they are proving to be a very effective method of modelling uncertain situations that depend on, or at least are assumed to depend on, cause and effect. Belief networks are especially useful when the information about the past and/or the current situation is vague, incomplete, conflicting, and uncertain. Aguilera *et al*. [19] provide a recent review of their use in environmental modeling, and note that even in this field their use is not yet widespread. Although Bayesian probability has long been well-known, the Bayesian propagation computations necessary, even for small belief networks, are very complex. It is only recently that efficient algorithms have been developed to compute propagation in networks of conditional probabilities with a reasonable number of variables, hence they are a relatively new applied analytical tool. 

**Table 1:** Policy objective and instrument priorities for improved animal welfare [20].

Policy Objectives (Columns) & Policy Instruments (Rows)	Pubic Awareness of AW (1)	On-Farm Standards (2=)	Chain Education (2=)	Off-Farm Standards (4)	Consumer Education (5)	Consumer Trust & Confidence (6)	Market Development (7)
**Government Regulations**	*3.1*	*3.7*	*3.5*	**4.0**	*3.0*	*3.4*	*3.2*
**Private Regulatory Standards**	*3.1*	*3.7*	*3.5*	*3.7*	*3.1*	*3.5*	*3.7*
**Incentive payments for farmers**	*2.8*	***3.9***	*3.6*	*3.7*	*2.7*	*3.1*	*3.6*
**Labelling (with 3rd party inspection**	**4.0**	*3.8*	*3.5*	*3.7*	***3.9***	**4.0**	**4.0**
**Private Incentives (without 3rd party inspection)**	*3.0*	*3.0*	*3.0*	*2.9*	*3.3*	*3.0*	*3.1*
**Education of General Public**	**4.4**	*3.7*	*3.5*	*3.6*	**4.4**	**4.0**	**4.0**
**Education/Training for Chain Actors**	*3.6*	**4.1**	**4.3**	**4.1**	*3.3*	*3.6*	*3.6*
**Increased Capacity Building**	*3.4*	*3.8*	**4.0**	*3.7*	*3.3*	*3.3*	*3.5*
**Promoting R&D**	*3.7*	**4.1**	3.9	**4.0**	***3.9***	*3.7*	*3.8*

As part of the EconWelfare project a major Delphi survey was conducted with stakeholder representatives across eight EU countries. Of the 458 people approached to take part in this survey, 194 full responses were obtained over the complete Delphi cycle. A major output of this survey was a policy matrix associating the most appropriate policy instruments to achieve a set of prioritized policy objectives. This matrix is shown in Table 1, with the policy objectives (column headings) ordered according to their relative importance as revealed in the survey, and average effectiveness of the major instruments (row headings) in achieving the respective objective recorded as the average score in each cell) on a scale of 1–5.

**Figure 3:**
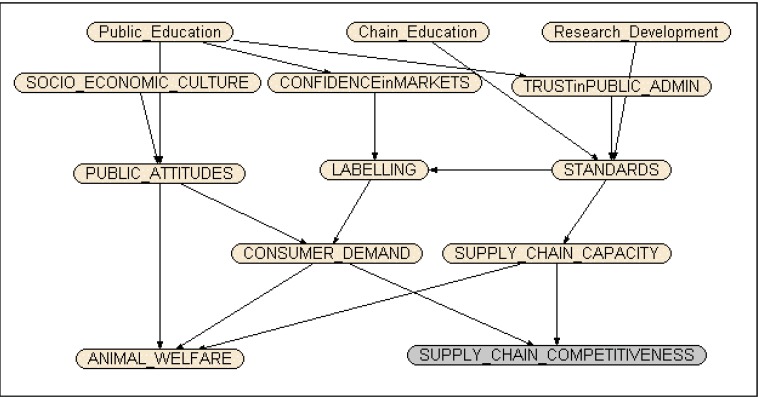
EconWelfare Belief Network (beta version).

Identifying the consequences of these generally preferred policies and instruments for the competitiveness of European agriculture is a tall order, and is plagued by both imprecision of the specific instruments and their effects, and about general uncertainty of the consequences. However, it is possible to propose an initial specification (called ‘alpha’ in the belief network literature) of the general causal relationships between these major instruments and policies based on the conceptual framework outlined in the previous section. An alpha version of the belief network was presented to 20 stakeholders (NGO, industry, academic and farmer representatives) at a workshop in March 2011. During this interactive workshop, stakeholders were asked to consider and amend—according to their animal welfare expertise and *beliefs—*the general structure of the model (the definition of nodes) and the direction of causality (edges) between the nodes. The amended alpha model was subsequently presented to the European Farm Animal Welfare Council meeting in Bergen in March, 2011 and the members were also asked to contribute to the improvement of this model. In addition, researchers involved in the Econ Welfare project have also contributed to the specification of the BBN representation. The result of these discussions was the final beta version (Figure 3).

This representation contains the key variables identified in the Delphi survey, and includes a preliminary judgment about the directional causation between them. At the centre of the belief network is *LABELLING*, the effectiveness of which depends on the underlying *STANDARDS*, which may be weak or strong. Although this particular instrument (Labelling) does not score especially highly in the Delphi survey, it is important for market development and is the fulcrum through which the other preferred instruments (on and off farm standards) are likely to have their effects on the priority objectives. The strength of the standards depends on three key factors, as identified here: the *Chain Education*, *R&D*, and *TRUST in PUBLIC ADMIN*, since the administration and implementation of standards clearly affects the strength of standard application. Trust in Public Admin, in turn, depends on *Public Education*. The effectiveness of Labeling also depends on *TRUST in MARKETS*, which also depends on the extent of Public Education, since the effectiveness of labels requires people to trust their provenance. Given well-informed and well implemented labels, backed up with strong standards, EU *ANIMAL WELFARE* then depends also on *PUBLIC AWARENESS*, conditioned by the *SOCIO-ECONOMIC CULTURE*, where *a priori* people in richer countries might be expected to be more aware of animal welfare as an issue, and willing to do something about it, than those in poorer countries.

Added to the variables from the Delphi are the Willingness to Pay (WTP) for AW (*CONSUMER DEMAND*) and the Willingness to Comply (*SUPPLY CHAIN CAPACITY*) variables. WTP is a demand-side measure of the extent to which consumers are willing to support improved animal welfare though paying a premium (as necessary) for high standard, labelled products. Supply Chain Capacity is a supply-side measure of the extent to which the supply chain, including farmers, has both the capacity and the willingness to provide better AW, and is expected to be greater the lower the net costs (greater the net benefit) of ensuring improved AW. These two variables combine to determine the effects of animal welfare standards and labels on the competitiveness of the EU animal production and supply chain (*SUPPLY CHAIN COMPETITIVENESS*). They also determine the level of ANIMAL WELFARE, in conjunction with public attitudes.

This beta model was circulated to all EconWelfare partners and sent to those previously responding to the Delphi survey. Experts were asked to provide as many 'cases' (collections of node ratings) as possible, and for each case to ‘score’ according to their judgment and beliefs the state of the current animal welfare system in their country, region or livestock sector. Each node included four ‘states’ with a score between 1 and 4 (e.g., for labelling: very effective = ‘1’, effective = ‘2’, ineffective = ‘3’ and very ineffective = ‘4’). 82 cases across the eight countries were received by the end of June 2011.

However, even this highly simplified picture of the EU Animal Welfare system still suffers from considerable complexity. As specified in the ‘beta’ version of the network, there are more than 800 conditional probabilities. To properly ‘train’ this network with expert judgments of the scores for each node would require upwards of 8,000 cases (separate judgments of present conditions for specific cases (countries and sectors)). Further simplification is both possible and necessary for our purposes. In this case, condensing the responses in each node from four possibilities (e.g., very high; high; low; very low) to two (‘high’ or ‘low’) substantially reduces the total conditional probability set to 84, which is more consistent with our data. However, we have been unable within the resources of the EconWelfare project to obtain enough separate judgments for a substantially reliable representation, so what follows can only be considered as an illustration of the approach.

**Figure 4:**
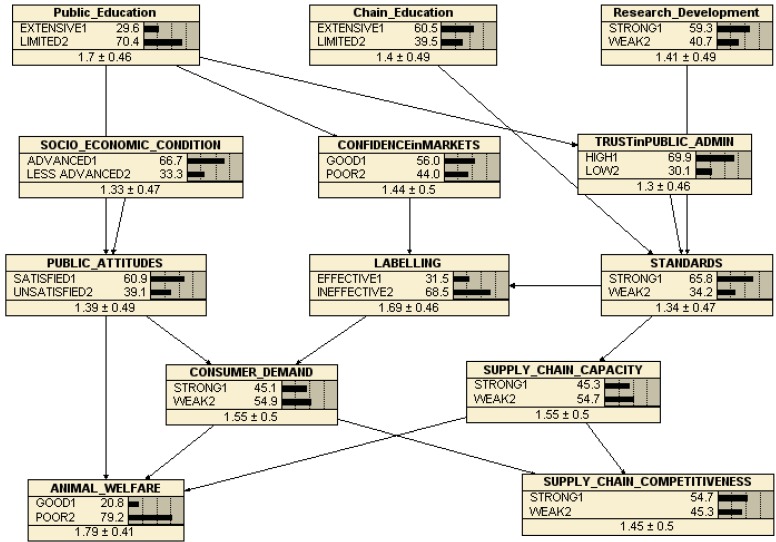
Calibrated EconWelfare Belief Network.

Our ‘trained’ (calibrated) version of the Belief Network is shown in Figure 4. Each variable (node) box shows the proportion of our total sample of cases (82) answering with each score. In each case, except the bottom left hand (outcome) node (ANIMAL WELFARE), the 4-scale scores have been contracted to 2-scale by aggregating the very good/good to the ‘good’ score and the poor/very poor scores to the ‘poor’ score. However, our respondents provided judgments about the present conditions of animal welfare in the EU which were highly skewed towards the good/very good end of the spectrum (a notable result in itself), with only 7% of our responses recording present animal welfare as poor or very poor. As a consequence, the trained network showed very little response of animal welfare to changes in any of the presumed ‘drivers’ in this case. However, re-calibrating the network to consider only the very good response (20.8%) in the animal welfare node as ‘good’, and treating all other responses (79.2%) as ‘poor’ allows some indicative response patterns to be identified.

The bi-value distribution of our respondents’ judgments on the current state of the variables driving animal welfare and supply chain competitiveness are shown in Figure 4. It is notable that our respondents tend to judge labelling and public education limited or ineffective, 68.5% and 70.4% respectively, rather than extensive or effective, while they are more satisfied with the current states of chain education, R&D, trust in public administration, standards and public attitudes.

**Figure 5:**
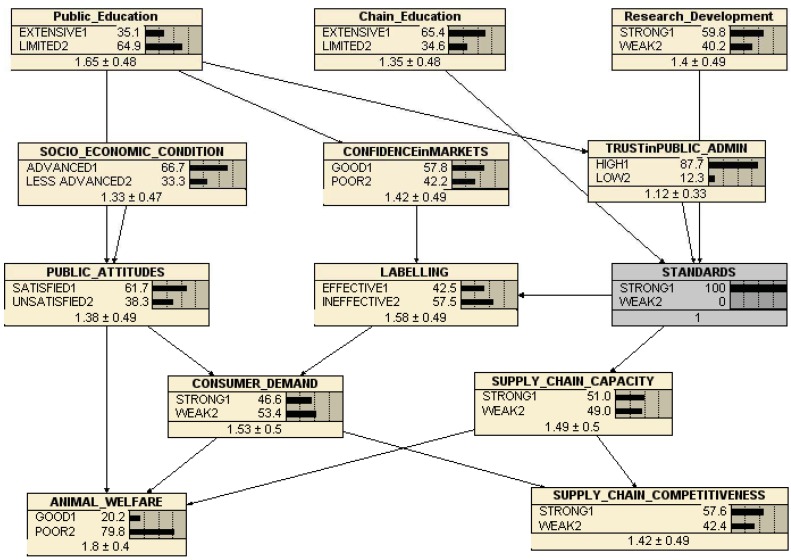
Network consequences of Improved Standards.

The calibration of the network with the set of 82 more or less different beliefs about the current states of each of the major factors (variables) allows the software [21] to construct a set of conditional probabilities which are implied by these different cases. As a result, it is now possible to use the network to illustrate the consequences of changing the state of the major drivers of animal welfare. For instance, Figure 5 shows the same network except that the state of *standards* is reset from the calibrated 65.8% strong to 100% strong. The consequences, according to the conditional probabilities implied by our respondents’ beliefs about the state of animal welfare and its major drivers in the EU, are that animal welfare actually declines marginally from being 20.8% good to 20.2% good, while supply chain competitiveness is slightly improved from 54.7% strong to 57.6% strong. This follows from associated improvements in supply chain capacity (from 45.3% to 51% strong) and labelling effectiveness (from 31.5% to 42.5%), and thus in consumer demand (from 45.1% strong to 46.6% strong). Hence, although the underlying conception of the behavior of the chain, which informs the structure of the belief network, might suggest that tighter/stronger standards would (other things equal) tend to reduce the competitiveness of the supply chains, the network captures at least some of the consequences of improved standards, which are that other things do not remain equal when changing one variable in a complex system. At least according to this network, however, improving standards is not sufficient to improve animal welfare on its own, since the positive effects via labeling and consumer demand are more than offset by an apparent negative consequence of improving supply chain capacity (see below).

**Figure 6:**
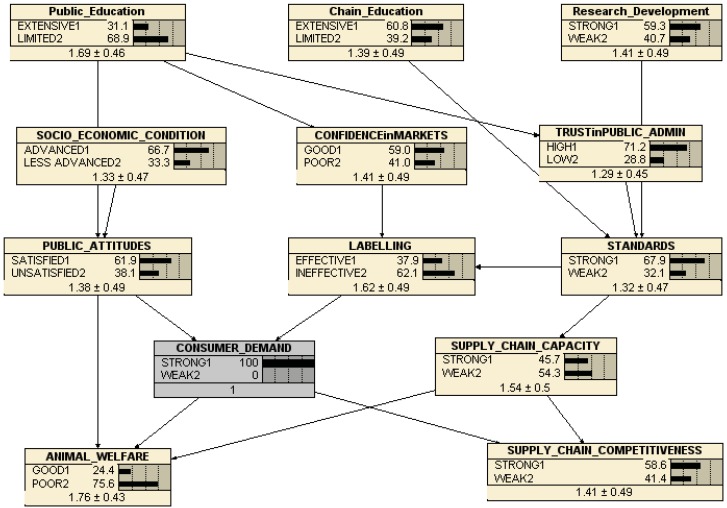
Belief Network consequences of improved consumer demand.

Similarly, Figure 6 shows the implications of improving consumer demand from 45.1% strong to 100% strong. If this could be achieved, the consequence would be both an improvement in animal welfare (20.8% to 24.4% good) and in supply chain competitiveness (from 54.7% to 58.6% strong). The implications, according to this belief network, are that the determinants of market demand, especially the effectiveness of labelling and the strength of standards, would also improve, from 31.5% to 37.9% effective and from 65.8% to 67.9% strong respectively.

The implications of this pattern of beliefs (noting the *caveat* that we do not have sufficient beliefs to calibrate, *i.e.*, train, this network robustly) are that improving animal welfare does not need to compromise supply chain competitiveness, despite the simple economic analysis of the PPF above, which apparently suggests that the twin objectives will generally be conflicting, other things being equal. The belief network shows that increasing consumer demand (increasing the relative value of higher animal welfare) is likely to both encourage the supply chain to deliver this higher value (resulting in improved animal welfare, and also to improve supply chain capacity as competitive pressures require the supply chain to become more responsive to this increased demand.

**Figure 7:**
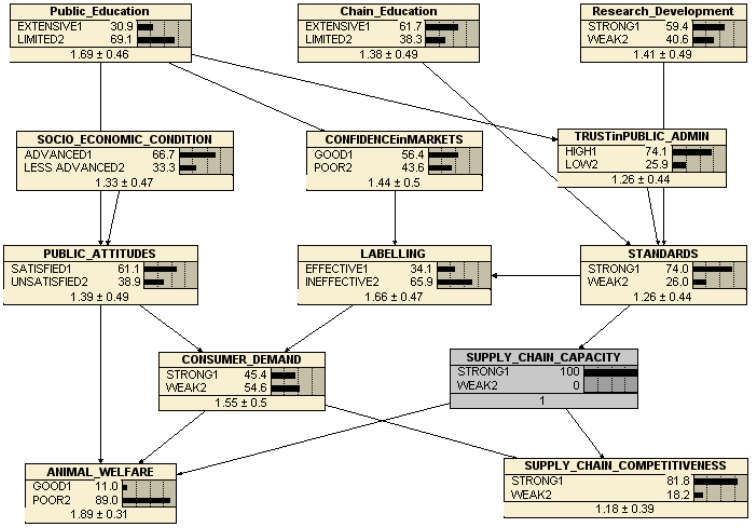
Belief Network consequences of improved supply chain capacity.

However, as Figure 7 illustrates, the belief that the objectives are in conflict is also apparent in this network. Figure 7 shows the consequences of improving supply chain capacity, according to the beliefs of our respondents and given this structure of causality. Improving supply chain capacity from 54.7% strong to 100% strong reduces animal welfare from 20.8% to 11% good, while improving competitiveness from 54.7% to 81.8%. This belief pattern apparently reflects the ‘vicious circle’ of animal welfare referred to in the introduction—that increased competition tends to harm animals as businesses strive to make money—rather than the ‘virtuous circle’ where more intelligent businesses pay attention to both actual and potential consumer and citizen demands, as well as more able producers learning that improved animal welfare can also be more productive, or at least no less productive. 

**Figure 8:**
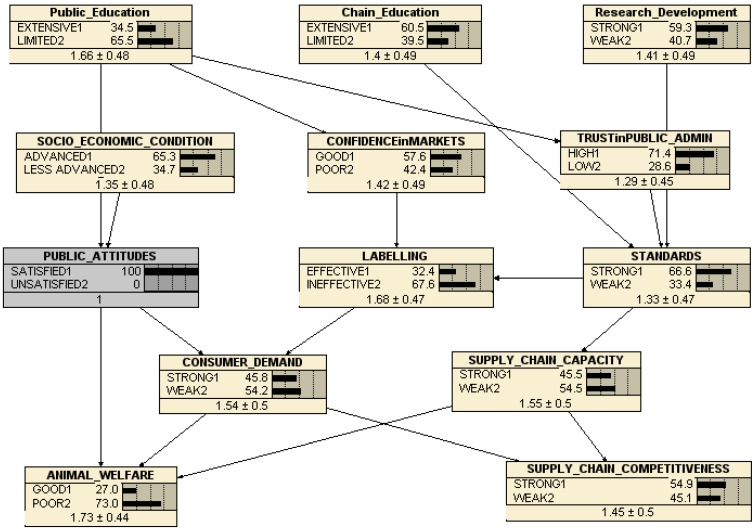
Belief network consequences of improving public attitudes.

Figure 8 shows the effects of improving public attitudes (revealed by our Delphi survey above as being the most important policy objective for improving animal welfare). This clearly improves animal welfare, from 20.8% to 27% good without compromising supply chain competitiveness (which does not change). However, the consequences of improving attitudes for the other major drivers (standards, labels, consumer demand, supply chain capacity) are all rather modest, though all improve. Improving public education is a major driver in this network for public attitudes, and improving this (not shown but available on request) does feed through the network to improve both animal welfare and supply chain competitiveness, again confirming the Delphi survey. However, while the survey strongly suggests that improving chain education and/or R&D should also be policy priorities, our prototype belief network does not demonstrate any substantial influence of these variables on either animal welfare or supply chain competitiveness.

## 5. Conclusions

Competitive supply chains, seeking to mobilize as much consumer spending as possible and operating within a single market, can be expected to deliver the levels of animal welfare that their customer segments are willing to pay for. Attempts to force the chains, by regulation, to improve standards above and beyond those which consumers are willing to support will tend to push animal production ‘off shore’, rather than improve home animal welfare conditions. Despite the common claim that animal welfare is a public good, and therefore the responsibility of government to ensure for all, closer analysis reveals that the potential market failure is really a free-rider problem, stemming from a psychological consumption externality (side effect)—my consumption of animal products affects your well-being. Overall animal welfare (number of animals times their individual welfare levels) is a private rather than a public good, determined by the consumption of animal products. Regulation of animal welfare, however, is a public good (or, *in extremis*, a public bad)—once provided and enforced for one it is supplied for all. Levels of animal welfare enforced by regulation result, essentially, from political competition between animal welfare advocates and stakeholders in the supply chains for public support, and result in levels of regulation that reflect these competing interests.

Nevertheless, there is every reason to suppose that there will always be a significant gap between citizens’ votes or opinions about animal welfare (necessarily elicited through questions focused specifically on the issues) and their willingness to pay as consumers to ensure these levels of animal welfare (expressed as part of the complexities of real lives). The gap between citizens’ votes and willingness to pay is not necessarily an indication of unfilled gaps in the market, which might be turned into markets in the gaps by competitive supply chains. Furthermore, the logic of the free-rider problem implies that the only problem-consistent policy strategy for its solution is a consumption subsidy for the consumption of more animal welfare friendly products, not a production or process subsidy as is commonly advocated. 

There is a need for governments to provide the genuine public good of reliable information—third party verification of standards, education and training of both the general public and the supply chain participants, definitions and measurement of animal welfare itself, and R&D to develop more animal welfare friendly production and treatment systems consistent with productivity. Given this provision, and the necessary common outlawing of unnecessarily ill-treatment of animals, markets (competitive supply chains) can be relied upon to progressively improve standards of animal welfare. This is demonstrated by both the comparative levels of animal welfare between countries and the history of animal welfare improvements over the recent past, as well as by the logic of the market process.

Finally, the Belief Network approach to identifying stakeholder judgments about the efficacy of improved animal welfare standards appears very promising as a route to exploring changing perceptions and attitudes towards improved animal welfare, and lubricating the all-important continued public debates and negotiations to that end.

## References

[1] Harvey D.R., Hubbard C. (2013). Reconsidering the Political Economy of Farm Animal Welfare: An Anatomy of Market Failure. Food Policy.

[2] Napolatino F., Girolami A., Braghieri A. (2010). Consumer Liking and Willingness to Pay for High Animal Welfare Products. Trends Food Sci. Technol..

[3] Keeling L.J., Immink V., Hubbard C., Garrod G., Edwards S., Ingenbleek P. (2012). Designing Animal Welfare Policies and Monitoring Progress. Anim. Welfare.

[4] McInerney J.P. Economic Aspects of the Animal Welfare Issue. Proceedings of the Meeting of Society for Veterinary Epidemiology and Preventive Medicine.

[5] Majewski E., Malak-Rawlikowska A., Gębska M., Gieldowska M., Spaltabaka E., Was A. (2011). Quantification of Farm Level Impacts of Introducing Upgraded Animal Welfare Standards for Selected Types of Farms.

[6] Franz A., von Meyer M., Spiller A. (2010). Prospects for a European Animal Welfare Label from the German Perspective: Supply Chain Barriers. Int. J. Food Syst. Dynam..

[7] Schmid O., Kilchsperger R. (2010). Overview of Animal Welfare Standards and Initiatives in Selected EU and Third Countries.

[8] Codron J.M., Giraud-Héraud E., Soler L.-G. (2005). Minimum Quality Standards, Premium Private Labels, and European Meat and Fresh Produce Retailing. Food Policy.

[9] Akerlof G.A. (1970). The Market for ‘Lemons’: Quality Uncertainty and the Market Mechanism. Quart.J. Econ..

[10] Eurobarometer (2007). Attitudes of EU Citizens towards Animal Welfare.

[11] Taylor D.H., Fearne A. (2006). Towards a Framework for the Improvement in the Management of Demand in Agri-Food Supply Chains. Supply Chain Manag..

[12] Mann S. (2005). Ethological Farm Programmes and the ‘Market’ for Animal Welfare. J. Agr. Environ. Ethics.

[B13-animals-03-00767] 13.Carlsson *et al* [14], find that, in the case of GM and GM free food products in Sweden, there is no significant difference between consumers’ wtp for a GM ban and for products labeled GM free. Since the ban overcomes the free-rider problem, while the wtp for labeled products ignores free-riding, in this case there is no apparent market failure. Nevertheless, free-riding is much more likely to be a major problem for environmental goods and services—which do have substantial public good elements, as, for instance, Hamilton *et al*., [15] explore.

[14] Carlsson F.R.C., Rykblom P.E.F., Lagerkvist C.J. (2007). Consumer Benefits of Labels and Bans on GM Foods—Choice Experiments with Swedish Consumers. Am. J. Agr. Econ..

[15] Hamilton S.F., Sunding D.L., Zilberman D. (2003). Public Goods and the Value of Product Quality Regulations: The Case of Food Safety. J. Public Econ..

[16] Ingenbleek P.T.M., Harvey D.R., Ilieski V., Immink V.M., de Roest K., Schmid O. (2013). The European Market for Animal-Friendly Products in a Societal Context. Animals.

[17] Harvey D.R., Hubbard C. (2011). Consequences of Imposing Upgraded Animal Welfare Standards through the Distribution Chain: A Socio-Economic Analysis.

[18] Harvey D.R., Hubbard C. (2011). Impacts of Improved Animal Welfare Standards on International Trade and Competitiveness of EU Animal Production.

[19] Aguilera P.A., Fernández A., Fernández R., Rumí R., Salmerón A. (2011). Bayesian Networks in Environmental Modelling. Environ. Model. Software.

[20] Hubbard C., Garrod G. (2011). Development of Policy Instruments and Indicators towards the Action Plan on Animal Welfare.

[B21-animals-03-00767] 21.We used Netica (Norsys) software, which “performs standard belief updating which solves the network by finding the marginal posterior probability for each node. … Netica assumes that conditional probabilities are independent and that prior probabilities are … continuous and bounded between 0 and1.” (Marcot *et al*. [22]).

[22] Marcot B.G., Steventon J.D., Sutherland D.G., McCann R.K. (2006). Guidelines for Developing and Updating Bayesian Belief Networks Applied to Ecological Modeling and Conservation. Can. J. Forest Res..

